# Special Issue: Next Generation DNA Sequencing

**DOI:** 10.3390/genes1030385

**Published:** 2010-10-27

**Authors:** Paul Richardson

Next Generation Sequencing (NGS) refers to technologies that do not rely on traditional dideoxy-nucleotide (Sanger) sequencing where labeled DNA fragments are physically resolved by electrophoresis. These new technologies rely on different strategies, but essentially all of them make use of real-time data collection of a base level incorporation event across a massive number of reactions (on the order of millions *versus* 96 for capillary electrophoresis for instance). The major commercial NGS platforms available to researchers are the 454 Genome Sequencer (Roche), Illumina (formerly Solexa) Genome analyzer, the SOLiD system (Applied Biosystems/Life Technologies) and the Heliscope (Helicos Corporation). The techniques and different strategies utilized by these platforms are reviewed in a number of the papers in this special issue. These technologies are enabling new applications that take advantage of the massive data produced by this next generation of sequencing instruments.

In this special issue, nine papers review and demonstrate the utility and potential of next generation sequencing. One of the biggest consequences with NGS technologies is how to deal with all of the data produced by these platforms. Magi *et al.* [1] review the software tools available for the multiple functions needed to process and interpret the huge amounts of data produced by these instruments. Sequence alignment to a reference, polymorphism detection, *de novo* assembly and visualization software are covered in this paper. Knudsen and colleagues [2] introduce a computer simulator that utilizes real and simulated reads to assess the effects of different factors and strategies for utilizing NGS to perform *de novo* assemblies. This is presently a particular challenge for the generally short (sometimes unpaired) reads produced by NGS Technologies. Another review article [3] on bioinformatic issues with NGS data focuses on statistical methods for analysis of Chip-Seq data as well as RNA data generated by these methods. NGS platforms are particularly suited to the use of ChIP-Seq methods as an alternative to ChIP-ChIP methods for identification of transcription factor binding sites. 

Another application that is particularly well-suited for the types of data produced using NGS is microRNA sequencing. MicroRNAs (miRNAs) and short interfering RNAs (siRNAs) are small RNA molecules of 17 to 24 bases that have been shown to play a critical role in gene regulation by mediating RNA interference. Motameny *et al.* [4] discuss in a review article the methods and strategies for capturing and sequencing miRNAs and their analysis. Epigenetic modifications, especially in the form of DNA methylation is another area of gene regulation that has recently gained significance in the research community. NGS platforms offer unprecedented abilities to elucidate the state of methylation in different species, individuals, tissues and cell types. Recent advances and strategies for characterizing the methylome are covered in two review articles [5,6] in this special issue.

The subjects of metagenomics (environmental sequencing) and sequencing of ancient DNA samples are two additional areas where NGS platforms have provided unforeseen opportunities for generating previously unattainable levels of understanding from these samples. Rooks *et al.* [7] demonstrate the utility of the 454 platform for assessing the viral ecology of a specific freshwater environment. Edwards *et al.* [8] utilized 454 technology on a marine microbial biofilm community to identify species and genes involved in cellulose degradation. Knapp and Hofreiter [9] review the progress and strategies for utilizing NGS platforms for generating genome sequence from ancient DNA samples, which are often contaminated with non-target DNA. The relatively inexpensive production of large amounts of data allows researchers to zero in on those sequences of most interest. Recent examples covered include woolly mammoth, Neanderthal and Palaeo-Eskimo. Finally, Anderson and Schrijver [10] review different NGS platforms and discuss the impact on the future of genomic medicine. 

The articles in this special issue represent a cross section of applications demonstrating the utility of NGS. New sequencing platforms are being introduced (next next gen, or third generation sequencing) by companies utilizing single molecule detection techniques to record individual DNA molecules during incorporation (Pacific Biosciences) or reading DNA directly by sieving it through nanopores. It is an exciting time in the field of genomics with rapidly changing and advancing technologies. The costs of sequence will surely continue to decrease and new applications of this technology will be introduced to address critical areas in academic and clinical research. I would like to thank all the authors and reviewers who contributed to this special issue and look forward to hearing about new sequencing technologies and application advances in the future.
